# Allopurinol reverses mercaptopurine-induced hypoglycemia in patients with acute lymphoblastic leukemia

**DOI:** 10.12688/f1000research.17760.2

**Published:** 2019-11-21

**Authors:** Melissa Zhang, Bruce Bostrom

**Affiliations:** 1 University of California, Berkeley, Berkeley, CA, 94720, USA; 2 Department of Pediatric Oncology, Children's Hospitals and Clinics of Minnesota, Minneapolis, MN, 55404, USA

**Keywords:** mercaptopurine, allopurinol, hypoglycemia, morning nausea, thiopurine methyltransferase

## Abstract

Fasting hypoglycemia is a known complication of mercaptopurine (6MP) maintenance therapy for acute lymphoblastic leukemia (ALL). It is associated with high levels of the methylated metabolite 6-methyl-mercaptopurine (6MMP). Symptoms of hypoglycemia include morning tremulousness, nausea and vomiting. We have previously shown that switching 6MP dosing from evening to morning resolved hypoglycemia by reducing 6MMP; however, the reduction of 6MMP was only transient, potentially resulting in return of hypoglycemia. In children and adults with Crohn’s disease, co-prescribing allopurinol with 6MP blocks the activity of thiopurine methytransferase (TPMT), reducing 6MMP and improving its tolerance. As a consequence of inhibiting TPMT, 6MP is shunted toward the production of 6-thioguanine nucleotide (6TGN), which will result in pancytopenia if the dose of 6MP is not reduced. We demonstrate that allopurinol with a reduced dose of 6MP in two patients with ALL and 6MMP-associated hypoglycemia resulted in a complete and sustained suppression of 6MMP and rapid reversal of hypoglycemia and its symptoms.

## Introduction

Mercaptopurine (6MP) maintenance therapy is critical for the cure of ALL. There is no acceptable alternative. In general, 6MP is well tolerated with minimal side-effects such as facial or generalized rash and asymptomatic elevations of hepatic transaminases. Occasionally, more serious side effects, such as direct hyperbilirubinemia, pancreatitis, and fasting hypoglycemia, may occur, requiring discontinuation or reduction in the dose of 6MP ^1^.

Previously, we had shown that 6MP induced fasting hypoglycemia is related to elevated levels of red cell 6-methyl-mercaptopurine (6MMP) ^2^. We also showed that altering the administration time of 6MP from evening to morning or splitting the dose to twice a day results in lower 6MMP concentrations and resolution of hypoglycemia symptoms. However, in some of the patients there was a rebound in 6MMP, which may result in recurrence of symptomatic hypoglycemia.

Allopurinol has been used by gastroenterologists for many years in patients with inflammatory bowel disease who have elevations of alanine aminotransferase (ALT) or gastrointestinal symptoms from the use of 6MP or azathioprine ^3– 6^. Recently, pediatric patients with ALL have been treated with allopurinol to reduce elevated 6MMP levels resulting in pancreatitis, hepatotoxicity, or inability to get absolute neutrophil count in target range despite increasing the dose of 6MP ^7– 9^.

## Case series

The electronic medical records of two children with ALL who developed symptomatic hypoglycemia on maintenance therapy were reviewed. After an extensive risk-benefit discussion with parents, they were started on allopurinol with a reduced dose of 6MP.

Thiopurine metabolites were measured with a CLIA-approved test ( www.prometheuslabs.com). The reference values for this assay only apply to patients with inflammatory bowel disease on azathioprine or 6MP and not for ALL patients on 6MP. Unpublished data on 200 patients with ALL from day 85 of the first maintenance cycle on Children’s Oncology Group COG1922 demonstrated the 5 ^th^, 50 ^th^, and 95 ^th^ percentiles for 6MMP are 320, 4900, and 19,000 pmol/ 8×10 ^8^ RBC, respectively. The 5 ^th^, 50 ^th^, and 95 ^th^ percentiles for 6TGN are 75, 260, and 690 pmol/ 8×10 ^8^ RBC, respectively ^10^. This two-patient case report was reviewed by Children’s Minnesota Institutional Review Board and deemed not research allowing publication.

### Case 1

Patient UPN 1 is an African-American girl who was diagnosed with B-lineage ALL at age 10 years. She was enrolled on high-risk protocol Children’s Oncology Group (COG) AALL1131 (ClinicalTrials.gov Identifier: NCT02883049), but was taken off protocol after induction due to desire to use triple intrathecal therapy for blasts in diagnostic cytospin (CNS-2 status). Germline testing for methylene tetrahydrofolate reductase (MTHFR C667T) and thiopurine methyltransferase (TPMT) were homozygous normal.

Maintenance therapy doses were started at 6MP (62 mg/m ^2^/day) and MTX (15 mg/m ^2^/week). On day 57 of maintenance, the dose of 6MP was increased to 75 mg/m ^2^/day with no change in the methotrexate (MTX) dose. On day 73, she presented to the emergency department with shaking. Upon questioning she disclosed having episodes of morning shaking, nausea and vomiting for about a month. Serum glucose was 3.18 mmol/L (53 mg/dL). The hemoglobin A1C level was 4.9%. Thiopurine metabolites showed an extremely elevated 6MMP level of 41,000 pmol/8×10 ^8^ RBC and 6TGN level of 456 pmol/ 8×10 ^8^ RBC. She was neutropenic with absolute neutrophil count (ANC) of 0.462 × 10 ^9^ cells/L so oral chemotherapy was halted.

**Figure 1.**
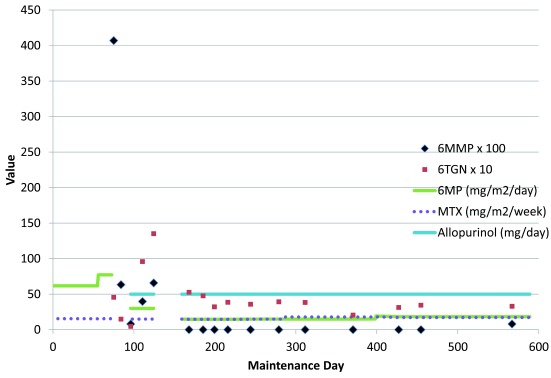
Treatment regimen for patient in Case 1. On the horizontal axis is the day of maintenance therapy from the start to completion. On the vertical axis are the red cell thiopurine metabolite values and drug doses. Interruption in drug doses is noted by a break in the line. For graph clarity, 6-methyl-mercaptopurine (6MMP) values were divided by 100 and 6-thioguanine nucleotide (6TGN) values by 10. After introduction of allopurinol, the 6MMP levels rapidly fell to undetectable levels with stable 6TGN. Following the initial introduction of allopurinol in patient 1 the mercaptopurine (6MP) and methotrexate (MTX) doses required interruption due to neutropenia which did not recur with a dose reduction of 6MP. As expected the myelosuppression was associated with a very elevated 6TGN level.

On day 98, 6MP was restarted at 30 mg/m ^2^/day along with 50 mg of allopurinol given with each dose of 6MP. MTX was also restarted at the previous dose. The episodes of morning nausea, vomiting, and shakes resolved. No further episodes of hypoglycemia were seen. On day 142, the ANC was 0.29 ×10 ^9^ cells/L, so oral chemotherapy was halted. Subsequently the hemoglobin level fell to 57 g/L and platelets to 82,000/µl. On day 163, 6MP was restarted at 15 mg/m ^2^/day with 50 mg allopurinol and the previous MTX dose, which continued to the end of therapy without interruption. MTX dose remained unchanged at 75 mg/m ^2^/week and 6MP was increased to 18 mg/m ^2^/day to keep ANC within the target range (0.5–2 ×10 ^8^/L). The patient remains in remission 24 months off therapy. Figure 1 contains details of oral chemotherapy doses and laboratory values.

### Case 2

Patient UPN 2 is a Caucasian girl diagnosed with B-lineage ALL at 3 years of age. Genotyping for TPMT was normal and MTHFR C677T was heterozygous. She was enrolled on the standard risk protocol COG AALL0932 (ClinicalTrials.gov Identifier: NCT01190930) and removed from the protocol when allopurinol was started.

Around day 124 of maintenance, she had problems with morning vomiting daily. She had been on full dose 6MP (75 mg/m ^2^/day) and MTX (20 mg/m ^2^/week) since the start of maintenance with no interruptions. On day 229, she was diagnosed steroid-induced hyperglycemia with rebound hypoglycemia. Hemoglobin A1C was normal. Home glucose monitoring was started. Glucose levels were noted to be elevated after completion of a 5-day dexamethasone pulse. Metformin 500 mg extended release every morning was started with a subsequent dexamethasone pulse on day 255, with the resolution of steroid induced hyperglycemia. However symptomatic hypoglycemia continued.

 Thiopurine metabolite levels were drawn, which showed an extremely high 6MMP level (32,718 pmol/8×10 ^8^ RBC) with a 6TGN level of 182 pmol/8×10 ^8^ RBC. She then was switched to morning dosing of 6MP, based on prior publication ^2^. She continued to have symptoms of morning hypoglycemia, which was confirmed on five low serum glucose values over a 40-day period (values of 46, 44, 42, 37, and 36 mg/dL = 2.8, 2.7, 2.6, 2.5, 2.2, and 2.2 mmol/L).

**Figure 2.**
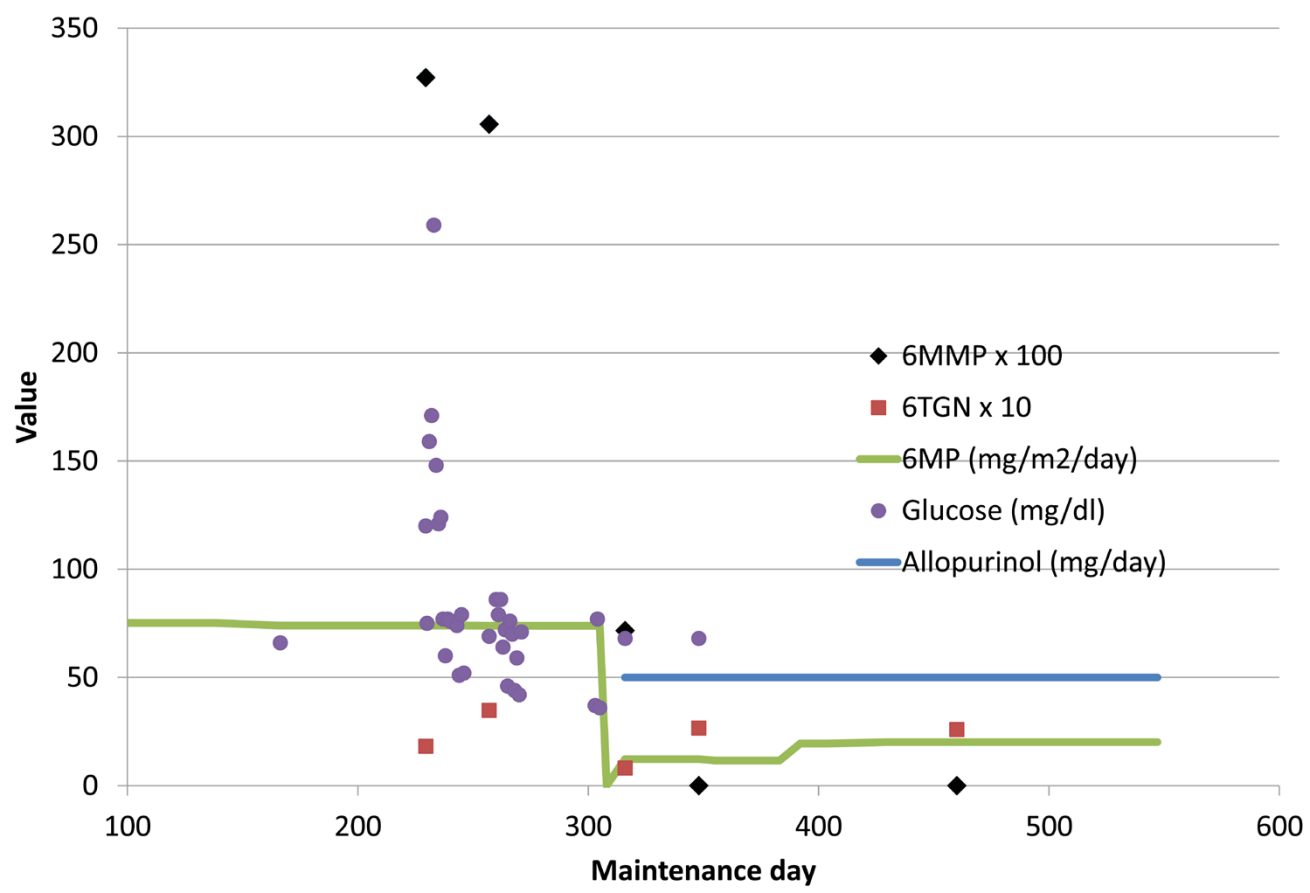
Treatment regimen for patient in Case 2. On the horizontal axis is the day of maintenance therapy from the start to completion. On the vertical axis are the red cell thiopurine metabolite values and drug doses. For graph clarity, 6-methyl-mercaptopurine (6MMP) values were divided by 100 and 6-thioguanine nucleotide (6TGN) values by 10. After introduction of allopurinol, the 6MMP levels rapidly fell to undetectable levels with stable 6TGN. Of note the high glucose values during dexamethasone pulses resolved after introduction of metformin on day 255.

After extensive discussion with the parents concerning the risks and benefits of the treatment, she was taken off COG 0932 protocol per physician preference and started on allopurinol 50 mg daily with reduced dose 6MP (12 mg/m ^2^/day) and MTX (11 mg/m ^2^/day) on day 316 of maintenance. Within 2 weeks she had no hypoglycemia symptoms and no low glucose values on home testing. On day 392 the doses were increased to 6MP (20 mg/m ^2^/day and MTX (17 mg/m ^2^/week) to keep ANC in the target range (0.5-2 × 10 ^8^/L). Metformin was continued for 5 months during dexamethasone pulses. Metformin was omitted the last 5 months of maintenance, without rebound hyperglycemia, which was completed on day 547. She remains in remission 24 months off therapy. Figure 2 contains details of oral chemotherapy doses and laboratory values.

## Discussion

6MP is a pro-drug that is metabolized in nucleated cells to form 6TGN, which is felt to be the active anti-leukemic metabolite. Alternatively, 6MP can also undergo oxidation through a pathway catalyzed by xanthine oxidase/dehydrogenase and aldehyde oxidase to form thiouric acid, which is excreted in urine. It is also metabolized in nucleated cells by TPMT, producing 6MMP. In patients with inflammatory bowel disease, high levels of 6MMP (>5300 pmol/8×10 ^8^ RBCs) have been associated with hepatotoxicity, decreased therapeutic efficacy, and symptoms of hypoglycemia ^11^.

Previously we showed that switching 6MP from evening to morning administration reduced elevated 6MMP levels and resolved symptomatic hypoglycemia ^2^. The mechanism of this effect is not fully known, but the most reasonable interpretation is inhibition of TPMT activity. The current study is an extension of that observation, showing that co-administration of a low dose allopurinol (50 mg) once a day with a reduced dose of 6MP (~20 mg/m ^2^/day) also resolves 6MMP-induced symptomatic hypoglycemia without any rebound of 6MMP levels, as we saw with switching the administration time of 6MP. A reduction in the dose of 6MP is needed because on average the 6MMP to 6TGN ratio during ALL maintenance is approximately 25 to 1, per unpublished data from the COG9506 study ^12^.

Approximately 90% of people, including the patients in this study, have wild-type *TPMT*, the genotype responsible for high levels of TPMT activity. These patients require higher doses of 6MP to reach therapeutic levels of 6TGN. It is unknown what proportion of these patients will develop the symptoms related to elevated 6MMP ^8^.

Allopurinol was first developed by Gertrude Elion to potentiate the therapeutic index of oral 6MP for treatment of leukemia. Albeit allopurinol stimulated the anti-tumor activity of 6MP, it was associated with increased hematologic toxicity. Allopurinol use with 6MP was abandoned in the 1960s and fell into the niche of managing gout. However, our data shows success of co-prescription of allopurinol to reverse hypoglycemia in children with ALL by reducing 6MMP ^13^.

Seinen *et al.* demonstrated that allopurinol inhibited xanthine oxidase/dehydrogenase and increased hypoxanthine guanine phosphoribosal transferase in blood samples from patients taking 6MP who were started on allopurinol ^14^. Notably, they did not show change in TPMT activity but did show a slight increase in 6TGN and significant decrease in 6MMP. However, Blaker *et al.* demonstrated inhibition of TPMT by a metabolite of allopurinol thioxanthine (2-hydroxymercaptopurine) *in vitro* ^11^. Our data shows that the same mechanism of allopurinol to inhibit TPMT to treat hepatotoxicity in IBD can be applied to reverse symptoms of hypoglycemia by lowering 6MMP levels. Both patients exhibited a reduction of 6MMP to undetectable levels following co-prescription of allopurinol with 6MP.

Of concern is the theoretical possibility that reducing production of 6MMP may have a negative effect on leukemia therapy. An *in vitro* study with MOLT-4 ALL cells showed that knocking down TPMT expression did not affect sensitivity to 6MP, and that increasing the 6MMP to 6TGN ratio in the MOLT-4 ALL cell line by adding S-adenosylmethionine (SAM) decreases cytotoxicity of 6MP ^15, 16^. These two prior studies suggest 6MMP is not an active metabolite in the treatment of leukemia. However, another *in vitro* study showed that transfection with *TPMT* gene increased sensitivity to 6MP in human CCRF-CEM cell lines, probably through inhibition of *de novo* purine synthesis by methylmercaptopurine nucleotide ^17^. To our knowledge, no studies on animals or humans, including currently unpublished results from the COG1922/B925 study, have demonstrated a correlation of intracellular levels of 6MMP with a decrease in ALL relapse. Thus, we are left with conflicting *in vitro* data and no patient data suggesting that 6MMP is necessary to cure ALL. Indeed, when prescribed by itself, allopurinol is also known to inhibit *de novo* purine synthesis, similar to the effect of 6MMP, suggesting it may have anti-leukemic effects. Following the submission of our manuscript the senior author reviewed for publication a two patient report further confirming our observations ^18^. In our opinion, the benefit of preventing symptomatic 6MMP-induced hypoglycemia, and the likely reduction or omission of 6MP, outweighs the unproven theoretical possibility of interfering with ALL therapy.

We recently became aware of the first reported use of allopurinol to modulate thiopurine therapy. They used a reduced dose of azathioprine as part of a combination immunosuppressive regimen for cadaveric renal transplant. The addition of allopurinol to the three drug combination of azathiopurine, cyclosporine and prednisolone reduced rejection episodes from 73% in historical controls to 8% without excessive bone marrow toxicity ^19^.

## Data availability

Deidentified clinical values for each patient by day are available on figshare. DOI: https://doi.org/10.6084/m9.figshare.7666409 ^20^.

Data are available under the terms of the Creative Commons Attribution 4.0 International license (CC-BY 4.0).

## Consent

Written informed consent for publication of their clinical details was obtained from the parents of the patients.
