# Supplementary material for: Allopurinol reverses mercaptopurine-induced hypoglycemia in patients with acute lymphoblastic leukemia
Source: F1000Res. 2019 Nov 21;8:176. Originally published 2019 Feb 11. [Version 2] doi: 10.12688/f1000research.17760.2 (PMC6392151; doi:10.12688/f1000research.17760.2)
Supplement: Peer review history

# Peer review: Reviewer response for version 1

**DOI:** 10.5256/f1000research.19418.r44317

**Published:** 2019-02-26

**Authors:** Michael E. Rytting

**Affiliations:** 1 Departments of Pediatrics and Leukemia, The University of Texas MD Anderson Cancer Center, Houston, TX, USA

This report on two patients with hypoglycemia and increased 6MMP provides an interesting discussion of management of this unusual but significant problem. The description of the use of allopurinol along with the biochemical rationale for using the drug is very helpful, and should be of value to pediatric hematologists. The discussion, including the possibility of altering the effectiveness of chemotherapy, is fair and balanced, as well.

I confirm that I have read this submission and believe that I have an appropriate level of expertise to confirm that it is of an acceptable scientific standard.

# Peer review: Reviewer response for version 1

**DOI:** 10.5256/f1000research.19418.r44316

**Published:** 2019-02-20

**Authors:** Maureen O'Brien

**Affiliations:** 1 Cancer & Blood Diseases Institute, Cincinnati Children's Hospital Medical Center, Cincinnati, OH, USA; 2 University of Cincinnati School of Medicine, Cincinnati, OH, USA

The authors provide an interesting report of the use of allopurinol to decrease 6-MMP levels and ameliorate 6MP-induced hypoglycemia. Their experience in these two cases is supported by other recent case reports with similar results. This article provides good background and discussion of the mechanism, as well as guidance regarding the risks of myelosuppression with this therapy and the need for 6MP dose reduction and close monitoring.  

 The authors should review the therapy details closely, particularly related to dosing. For patient #1 they state that the methotrexate dose was 75mg/m ^2^ which is clearly a typo, but draws into question if other doses provided are correct.

I confirm that I have read this submission and believe that I have an appropriate level of expertise to confirm that it is of an acceptable scientific standard.
